# Emergence, climate-driven expansion, and diversification of a European *Vibrio vulnificus* lineage (L4) with multi-host pathogenic potential

**DOI:** 10.1080/22221751.2025.2601370

**Published:** 2025-12-09

**Authors:** Héctor Carmona-Salido, Rubén Salvador-Clavell, Claudia Jäckel, Isabelle Schulze, Karla J.F. Satchell, Jens Andre Hammerl, Carmen Amaro

**Affiliations:** a Instituto Universitario de Investigación en Biotecnología y Biomedicina, Universitat de València, Burjassot, Spain; b Consultant Laboratory for Vibrio spp. in Food, Division Diagnostics, Pathogen Characterisation, Parasites in Food, Department Biological Safety, German Federal Institute for Risk Assessment, Berlin, Germany; c Department of Microbiology-Immunology, Northwestern University Feinberg School of Medicine, Chicago, Illinois USA

**Keywords:** *Vibrio vulnificus*, MARTX, European lineage, zoonosis, seafood safety, climate change

## Abstract

Climate-driven changes are reshaping the ecology of *Vibrio vulnificus* in European waters. Here, we present a retrospective genomic and phenotypic analysis of pre-2018 isolates belonging to lineage 4 (L4), a phylogenetic group historically confined to the Mediterranean Sea and now detected in northern Europe. Using a lineage-specific multiplex PCR combined with whole-genome sequencing, we identified 49 clinical and environmental L4 isolates from German coastal waters. Comparative genomics revealed extensive genetic plasticity in L4, indicative of frequent recombination and horizontal gene transfer, including three MARTX toxin architectures, fourteen distinct capsular genotypes, two type VI secretion systems, and multiple prophages. Notably, nearly half of the L4 isolates encoded a previously undescribed MARTX variant (type H), apparently derived from recombination within a type C toxin and containing a novel calmodulin-dependent NADase (CdN) domain with potential functional implications for virulence. One strain also harboured the plasmid-borne genes *ftbp* and *fpcrp*, which confer resistance to fish innate immunity and the ability to cause sepsis, thereby extending the distribution of the *piscis* pathovar to all five *V. vulnificus* lineages. Functional assays showed that most L4 strains withstood the bactericidal activity of iron-overloaded human serum, consistent with a capacity to cause sepsis in susceptible individuals. Collectively, these findings redefine *V. vulnificus* as a multi-host climate-responsive marine pathogen and establish L4 as a newly adapted European lineage whose northward expansion exemplifies how genomic diversification and ocean warming jointly drive the evolution of high-risk marine pathogens within a One Health framework.

## Introduction

*Vibrio vulnificus* is a highly virulent, multi-host marine pathogen that inhabits brackish-water ecosystems in tropical and subtropical regions [1]. It can cause fulminant sepsis in humans within 24–72 h of infection, particularly in individuals with underlying conditions that increase iron availability in the blood, such as liver disease or hemochromatosis [1,2]. Among these risk groups, the case-fatality rate can exceed 50%, even with antibiotic therapy and intensive care [2,3].

Human infections typically occur through two main routes: (i) exposure of wounds or damaged skin to seawater and (ii) ingestion of raw or undercooked seafood, particularly filter-feeding shellfish such as oysters or raw fish used in sushi and sashimi [1,4]. Because filter feeders concentrate *Vibrio* cells, they represent a well-documented source of infection [1]. In addition, a third, less common route has been documented – direct transmission from infected or carrier fish to humans, particularly among individuals handling diseased fish in aquaculture facilities or fish markets [5]. These cases provide clear evidence of zoonotic transmission in *V. vulnificus*.

Phylogenomic analyses based on core-genome variation have revealed five major lineages (L1–L5) within the species, each comprising environmental and clinical strains, along with a polyphyletic pathovar capable of causing sepsis in fish (pathovar *piscis*, pv. *piscis*) present in most lineages [6,7]. Lineages L1 and L2 are globally distributed, whereas L3, L4, and L5 are geographically restricted to the Mediterranean region. Specifically, L3 and L5 occur along the eastern Mediterranean coasts of Israel, while L4 has thus far been confined to the western Mediterranean, particularly the Spanish coast.

Strains of pv. *piscis* carry two accessory genes – *ftbp* (fish transferrin-binding protein) and *fpcrp* (fish phagocytosis and complement-resistance lipoprotein) – that confer species-specific resistance to the innate immune response in fish blood [8,9]. Both genes are located on a virulence plasmid (pVir) that can be horizontally transferred [10,11]. Remarkably, pv. *piscis* strains tested to date can also cause sepsis in mice, the experimental model for human vibriosis [12], and each pathovar group includes at least one human clinical isolate [6,11]. Together with documented cases of fish-to-human transmission [5], these findings support the view that *V. vulnificus* is a multi-host pathogen with zoonotic capacity.

Pathovar *piscis* has been detected in four of the five lineages, leaving L4 as the only group in which such strains had not yet been identified [11]. To date, L4 remains a minor lineage, represented by only two strains – one clinical and one environmental – both related to the Catalan coast of the western Mediterranean [6,13]. For this reason, it was originally considered the only lineage endemic to Europe.

**Figure 1.**
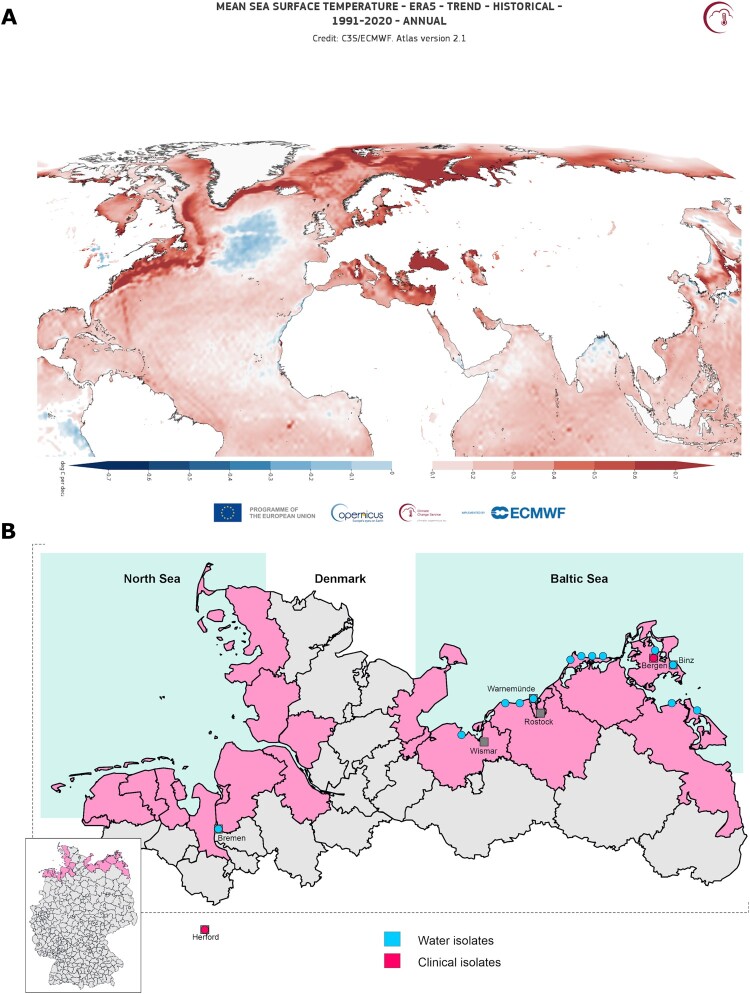
Maps of mean Sea Surface Temperature (SST) trend and subsequent *V. vulnificus* L4 isolate reporting. (A) Mean SST trend from 1991 to 2020 for the North Atlantic, European Seas and surrounding areas. The map shows the SST trend based on the ERA5 dataset. Red shading indicates warming trends, and blue shading indicates cooling trends (in °C). (B) Geographical distribution of clinical and environmental *V. vulnificus* isolates in Germany. Environmental isolates are represented with blue dots and clinical isolates with red dots.

The incidence of *V. vulnificus* infections in both animals and humans rises sharply during warm months and correlates closely with increasing sea surface temperatures (SST) [14,15]. Consequently, its prevalence has grown substantially in recent years as a result of global warming [14,16]. The current situation is alarming: since February 2023, SST have persistently exceeded 1.5℃ above pre-industrial levels, driven in part by El Niño conditions [17], surpassing even the most pessimistic projections. The sustained warming is especially notable in the northern European coastal areas, which have experienced an SST increase of approximately 0.7℃ over the last 20 years (Figure 1(A)). This warming has expanded the habitable range of *V. vulnificus* into previously unaffected northern European coasts [14,18].

The Baltic region exemplifies this growing concern: over 1,000 *Vibrio*-related infections were reported between 2014 and 2018, with further increases observed in recent years [19]. The European Food Safety Authority (EFSA) has emphasized that climate-driven warming of coastal waters is expanding the ecological niches of *Vibrio* species, thereby increasing infection risk across Europe [20].

In this study, we report that lineage L4 of *V. vulnificus*, historically confined to the Mediterranean, has now been detected in northern European waters. Using a newly developed lineage-specific multiplex PCR in combination with whole-genome sequencing, we analysed 221 clinical and environmental isolates from Germany and Spain and identified 49 new L4 isolates, all originating from Germany prior to 2018. Comparative genomics revealed extensive genetic diversity within L4, including three multifunctional-autoprocessing repeats-in-toxin (MARTX) toxin types – one of them novel and containing a previously undescribed domain arrangement – multiple capsular genotypes, two distinct type VI secretion systems (T6SS), and numerous prophages. Phenotypic assays further suggest that L4 strains can resist the bactericidal activity of iron-overloaded human serum and may cause sepsis in susceptible individuals. Remarkably, one L4 strain was identified as belonging to *pv. piscis*, thereby extending the distribution of this pathovar to all five phylogenetic lineages of the species.

## Material and methods

### Bacterial isolates and culture conditions

**Figure 2.**
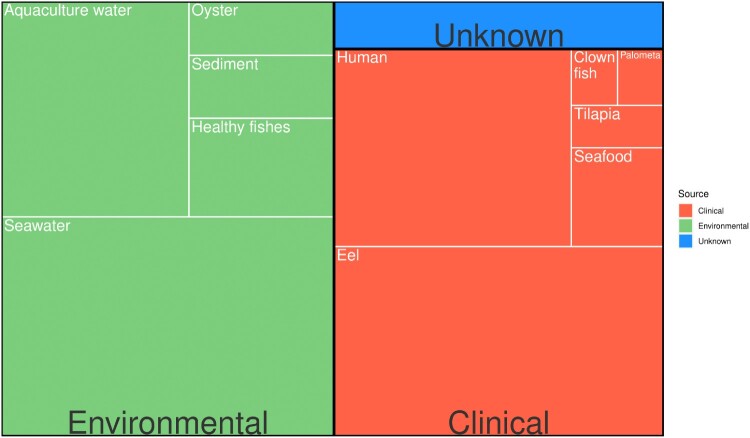
Treeplot of *V. vulnificus* sources used for L4 PCR validation (*n* = 222). Most strains were isolated from environmental or healthy organisms. The rest were isolated from clinical samples from humans or fish. Percentages in pie charts correspond to the total of strains whereas percentages in doughnut correspond to each category.

A total of 166 Spanish strains (Dr. Amaro’s collection, University of Valencia) and 55 German strains (DVG–Consultant Laboratory for *Vibrio* spp. in Food; German Federal Institute for Risk Assessment) from environmental and clinical sources were used in this study (metadata in Figure 2 and Supplementary Table 1). Sampling locations are shown in Figure 1(B). All isolates were collected between 1976 and 2017 and stored at −80℃ in LB-1 broth (Luria–Bertani supplemented with 1% NaCl and 20% glycerol). Routine culture was performed on tryptic soy agar supplemented with 0.5% NaCl (TSA-1) at 28℃ with shaking (180 rpm). Growth conditions were identical for all assays.

### Genomic dataset and phylogenetic analysis

To identify lineage-specific markers, all *V. vulnificus* reads available in the NCBI Sequence Read Archive (SRA, https://www.ncbi.nlm.nih.gov/sra/) as of February 2019 were retrieved, excluding incomplete or transcriptomic datasets. For all bioinformatic analyses, default parameters were used except where stated otherwise. De novo assemblies were produced with SPAdes v3.13.0 [21] in *careful* mode (–careful option), and quality was assessed with QUAST v5.0.2 [22] with “ – gene-finding” option (see Supplementary Methods). Genome assemblies with abnormal GC content (outside 45.2–47%) were excluded. The final dataset comprised 295 *V. vulnificus* genomes (Supplementary Table 2). Annotation was performed with Bakta v1.8 [23], and phylogenomic reconstruction was conducted using Parsnp v1.5.6 [24] with the closed genome of the pv. *piscis* strain CECT4999 as reference. The resulting tree was visualized with iTOL [25].

### Pangenome analysis and identification of an L4-specific marker

Orthologous gene clustering was performed using PIRATE v1.0.4 [26] with nucleotides search (-n option) and default clustering parameters. Strains clustering with previously defined L4 genomes [6] were assigned to L4. Among the nine core genes unique to L4, one encoding an ABC transporter permease was selected as a diagnostic marker based on its size, annotation, and conserved genomic context. This gene was then used for primer design and validation in the lineage-specific multiplex PCR.

### Multiplex PCR for L3 and L4 identification

A multiplex PCR assay was designed to simultaneously amplify *vvhA* (species marker) [27], *pgiA* (L3-specific marker) [28], and the L4-specific ABC-permease gene identified above. These three targets enable the differentiation of the Mediterranean lineages L3 and L4. Primer sequences, PCR conditions, and validation data are provided in the Supplementary Methods and Supplementary Table 3.

Genomic DNA was extracted from 221 isolates using the boiling method [29], and multiplex PCR products were resolved on 2% agarose gels containing GelRed (Biotium). A 100 bp GeneRuler Plus DNA ladder (Thermo Fisher Scientific) was used as molecular marker. Positive and negative controls were included in each run. Validation against whole-genome sequencing (WGS) confirmed 100% concordance between PCR-based lineage assignment and phylogenomic clustering.

### Whole-genome sequencing and lineage confirmation

All PCR-confirmed L4 isolates were sequenced on an Illumina MiSeq platform using 2 × 250 bp paired-end reads prepared with the Nextera XT DNA Library Kit. The sequencing depth ranged from 40 to 60. Assemblies were generated with the Shovill/Aquamis pipeline, and assembly quality metrics (coverage, N50, contig count, GC%) were determined with QUAST. Lineage confirmation and annotation was carried out with a phylogeny in Parsnp and Bakta respectively as described above (see Genomic dataset and phylogenetic analysis section and Supplementary Methods).

### Comparative genomic analysis

Virulence and antimicrobial resistance genes were annotated with Bakta, which integrates the Virulence Factor Database (VFDB) and the Comprehensive Antibiotic Resistance Database (CARD) [30]. Known virulence determinants – including *rtxA1*, capsule loci, and T6SS – were identified with Bakta, while antibiotic resistance genes were detected using ABRicate with minimum gene coverage and identity of 70% (https://github.com/tseemann/abricate). Protein sequences from M06-24/O (VVMO6_03947, NCBI Accession https://www.ncbi.nlm.nih.gov/protein/ADV88969.1/, [31]) and CMCP6 (VV2_0479, NCBI Accession https://www.ncbi.nlm.nih.gov/protein/AAO07430.1, [32]) were aligned with new MARTX sequence in Clustal Omega (http://www.clustal.org/omega/) and effector domains were manually annotated in MacVector 18.2.5 based on literature. Percent identity of conserved fragments (E-value < 10) was determined using NCBI BLASTP. Capsule loci were analyzed likewise using CPS-1 (*V. vulnificus* CMCP6), CPS-2 (*V. vulnificus* YJ016), and CPS-4 (*V. vulnificus* CECT4999) as references [33,34]. Visual comparisons of genomic regions were generated using Easyfig v2.2 [35]. The presence of prophages and plasmids was assessed using geNomad v1.6 [36], and fish-associated virulence genes (*ftbp*, *fpcrp*) were detected by HMM-based searches using reference sequences described by [11].

### Serum resistance assays

Commercial human serum (Sigma-Aldrich, lot H4522; pooled from >10 donors) and eel serum (pooled from five donors; provided by Valenciana de Acuicultura SA) (ES) were tested for sterility prior to use by plating 100 µL aliquots on TSA-1 and incubating at 37℃ (human) or 28℃ (eel) for 72 h. Randomly selected L4 strains, together with the serum-resistant control strain CECT 4999 (Table 1), were grown to stationary phase in LB-1 broth, diluted to 10⁴ CFU mL^−^¹ in serum (with or without 100 µM FeCl₃ added to simulate the main risk factor predisposing to sepsis in *V. vulnificus* infections [9]), and incubated for 4 h at the corresponding temperature. The incubation period was chosen based on previous in-house assays indicating a marked decline in bactericidal activity after approximately 4 h of incubation. Internal negative controls (serum-sensitive strains) were included in all experiments. Bacterial counts were determined by drop plating on TSA-1 at 0 and 4 h, and survival rates were expressed as the percentage of viable cells relative to the initial inoculum [24]. The isolates tested and their corresponding CPS genotypes are listed in Table 1.

**Table 1.** Resistance to bactericidal action of human serum of selected L4 strains with different capsular genotypes, with and without exogenous iron.

Strain	Capsular genotype*	Resistance**	
		Human serum	Human serum + iron
18-VB00126	5	± (85.4%)	NT
18-VB00194	5	± (65.4%)	NT
21-VB00144	12	± (94.6%)	NT
21-VB00145	12	± (76.2%)	NT
21-VB00147	12	+ (125%)	NT
10-VHB0198	11	+ (127.4%)	+++(42,769%)
20-VB0250	10	− (0%)	0
19-VB00243	FG	− (0%)	0
94385	8	+ (153%)	+++ (81,726%)
Riu1	17	± (72.8%)	+++ (41,994%)
**CECT4999 (Control strain)**	4	± (64.7%)	+++(218,703%)

*. Capsular genotype in Supplementary Figure 2 and Supplementary Table 3. **. Resistance to human serum: Values in parentheses represent the average percentage of survival from three independent experiments. Strains were incubated in pooled human serum at 37°C for 4 h, with or without exogenous iron (100 μM FeCl₃). Resistance was categorized as follows: –, survival rate between 0–60% (sensitive); ±, 61–100% (resistant); +, 101–999% (resistant and proliferative); ++, 1,000–9,999%; (resistant and marked proliferation); +++, ≥10,000% (resistant and highly proliferative). FG: refers to fragmented genomes excluded from comparative analyses due to incomplete assembly of key genomic regions. NT: not tested.

### Antimicrobial susceptibility testing

Antibiotic susceptibility was determined by disk diffusion on Mueller–Hinton agar containing 1.5% NaCl, following EUCAST 2024 standards [29]. The antibiotic panel included β-lactams, aminoglycosides, quinolones, and tetracyclines (full list and concentrations in Supplementary Table 4 and Supplementary Methods). *Escherichia coli* ATCC 25922, *Staphylococcus aureus* ATCC 25923, and *Pseudomonas aeruginosa* ATCC 27853 were used as quality controls. All L4 isolates tested were collected before 2018, and results are presented descriptively as “susceptible to frontline antibiotics,” avoiding overinterpretation in an epidemiological context.

### Integration of phenotypic and genomic data

Functional assays – including serum resistance and antimicrobial susceptibility – were used to validate genomic predictions and assess the clinical and ecological relevance of L4 isolates. The integration of comparative genomics and phenotypic data provided a comprehensive framework to evaluate L4 virulence, antibiotic susceptibility, and environmental adaptation under a One Health perspective.

## Results

### PCR design for L4 strain identification

**Figure 3.**
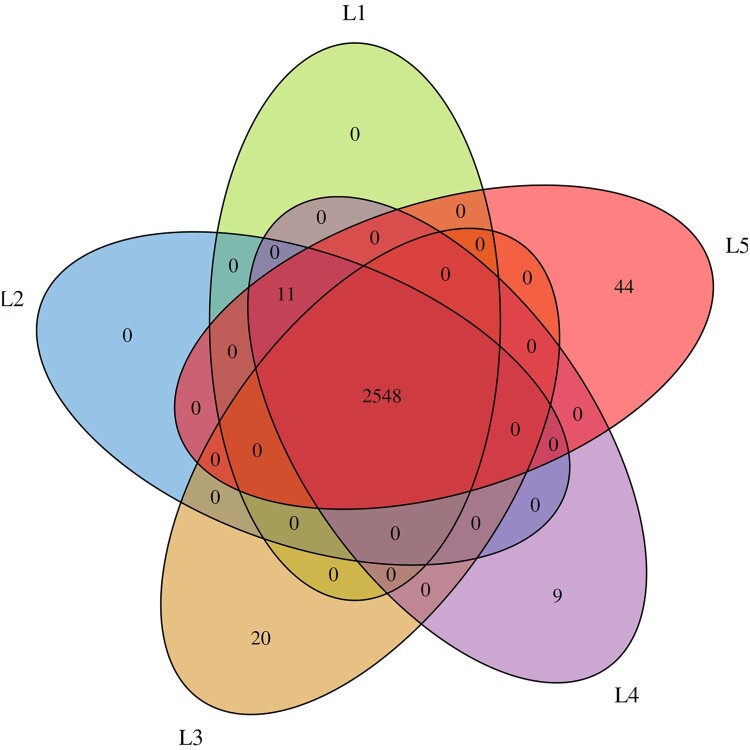
Venn diagram of the gene families of *V. vulnificus*. The number indicates the number of genes shared by all strains contained for each cluster.

To develop a molecular tool for the identification of L4 strains, we first performed a comparative pangenome analysis using 295 curated *V. vulnificus* genomes. Orthologous gene clustering with PIRATE yielded 21,113 gene clusters, of which 2,548 (12%) were shared across all strains (strict core genome). Within the L4 subset, nine core genes unique to this lineage were identified (Figure 3). Table 2 lists the annotations of these genes in the reference strain Riu1, along with their five upstream and downstream neighbouring genes. Among them, a gene encoding an ABC transporter permease was selected as the most suitable diagnostic marker based on its annotation, length, and conserved genomic context.

**Table 2.** Gene annotation of the Riu1 genomic region containing the nine L4 core genes identified by pangenome analysis, along with five upstream and five downstream neighbouring genes. Genes conserved across all tested L4 strains are shown in bold, and the gene selected for lineage-specific PCR assay design is *underlined*.

Gene	Length	Bakta annotation	Blastp annotation
*glgX*	2013	Glycogen operon protein GlgX	
	1809	hypothetical protein	GGDEF domain-containing protein
	1197	hypothetical protein	GGDEF domain-containing protein
	276	hypothetical protein	hypothetical protein
*yhhW*	852	Quercetin 2,3-dioxygenase	
	1422	***hypothetical protein***	*ABC transporter permease*
	693	**putative ABC transporter ATP-binding protein**	
	705	hypothetical protein	DUF3299 domain-containing protein
	726	**hypothetical protein**	DUF3299 domain-containing protein
*dan_1*	1644	**D-aminoacylase**	
*per1*	999	Extended-spectrum beta-lactamase PER-1	
*Dag*	1650	**N-acyl-D-glutamate deacylase**	
* *	1770	**Cardiolipin synthase**	
* *	915	**hypothetical protein**	Permease/DUF2959 domain-containing protein
*mdtN_1*	1059	Multidrug resistance protein MdtN	
* *	309	**hypothetical protein**	hypothetical protein
*ppnP*	282	Pyrimidine/purine nucleoside phosphorylase	
	510	hypothetical protein	DUF2850 domain-containing protein
*chiA_1*	2538	Chitinase A	
*menH_3*	849	2-succinyl-6-hydroxy-2,4-cyclohexadiene-1-carboxylate synthase	
	594	hypothetical protein	TetR/AcrR family transcriptional factor

**Figure 4.**
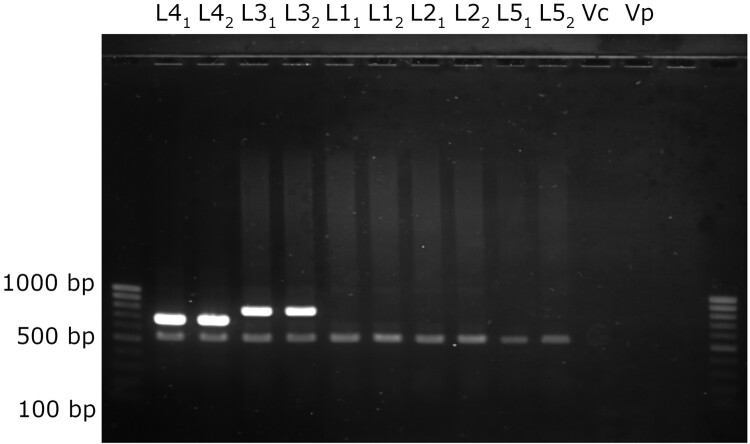
Multiplex PCR for L3, L4 and species identification. The PCR targets the species-specific gene *vvhA* (amplified fragment; 519 bp), the *pgiA_L3_* (amplified fragment; 834 bp) and the L4 gene (ABC, amplified fragment; 676 bp). First two lanes are L4 positive control strains (94385 and Riu1), next two lanes correspond to L3 positive control strains (12 and 11028), next 6 strains are *vvhA* positive control strains (L1 strains: yb158, YJ016; L2 strains: ATCC29307 type strain and CECT4999; L5: V252 and yb95). Two negative controls were used: *Vibrio cholerae* and *Vibrio parahaemolyticus*. First and last lanes are size markers.

A multiplex PCR was then designed for the simultaneous detection of *V. vulnificus* L4 and L3, the two lineages isolated from the Mediterranean Sea. The assay targets three genes: (i) *vvhA* (species marker), (ii) the L4-specific *ABC permease* gene, and (iii) the L3-specific *pgiA_L3* gene [28]. Primer sequences and expected amplicon sizes are shown in Supplementary Table 3, and PCR conditions are detailed in the Supplementary Methods. The assay was validated using the two previously known Spanish L4 isolates, with L3 and non-L4 isolates serving as negative controls (Figure 4).

### Phylogenetic and PCR identification of new L4 isolates

The multiplex PCR was applied to 55 German and 166 Spanish isolates from clinical and environmental sources, collected between 1976 and 2017 (Figure 4, Supplementary Table 1). The L3 primers amplified only the eastern Mediterranean control strains, while the L4 primers produced amplicons for 49 German isolates. All PCR-positive isolates were then subjected to whole-genome sequncing. Assembly statistics are shown in (Supplementary Table 5). PCR results were confirmed by phylogenomic analysis including the 295 curated *V. vulnificus* genomes. Core-genome SNP phylogenetics resolved the five major lineages (L1–L5) previously described [6]. All putative L4 isolates clustered unambiguously within the L4 clade, previously restricted to Spain. The complete phylogeny and metadata are shown in Supplementary Figure 1 and Supplementary Table 2. PCR and whole-genome results were fully concordant (100% specificity and sensitivity; Supplementary Table 1), confirming that the multiplex PCR provides a robust, lineage-specific tool for rapid identification of L4 isolates in surveillance programmes.

These results demonstrate that L4 is not confined to the Mediterranean but has dispersed into northern European coastal environments. Although all isolates predate 2018, they provide a clear retrospective signal of climate-linked northward distribution, consistent with the broader environmental expansion of *Vibrio* populations [14,19].

### Virulence and survival factors in L4 isolates

**Figure 5.**
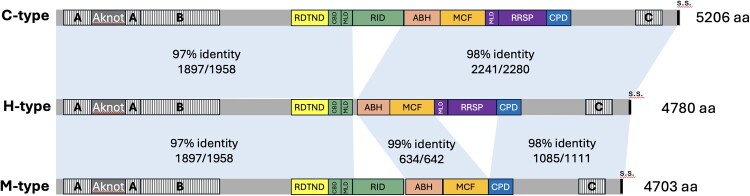
Domain architecture of MARTX toxin variants identified in *V. vulnificus* L4 on the basis of *rtxA1* gene variability. The figure compares the three MARTX variants identified: Type M from representative strain M06-24/O (NCBI ADV88969.1), Type C from representative strain CMCP6 (NCBI AAO07430.1), and the newly identified variant H from representative strain 94385. Each variant includes conserved N-terminal and C-terminal A, B, and C repeat regions, a C-terminal secretion signal (s.s.), an Aknot region for host cell glycan binding [54], and an autoprocessing cysteine protease domain (CPD), all involved in toxin secretion and effector translocation. The variable central region contains distinct effector domains: **RDTND** (RID dependent transforming NADase domain), that cleaves NAD^+^ essential for reactive oxygen species generation; **RID** (Rho Inactivation Domain), inactivates Rho GTPases to suppress proinflammatory gene expression; **ABH** (Alpha/Beta Hydrolase), a phospholipase A1 that blocks autophagy by targeting PI3P; **MCF** (Makes Caterpillars Floppy-like), triggers apoptosis and mitochondrial damage by targeting Rab GTPases; **RRSP** (Ras/Rap1-specific endopeptidase), and cleaves Ras/Rap1 GTPases, interfering with MAPK signaling. Both RRSP and RID have characterized membrane localization domains (MLD) and RID has a calmodulin binding domain (CBD). BLASTP amino acid identity among the sequences are shown in shades of blue (63%–100%). The new variant reconfigures domain architecture by removal of the RID catalytic domain sequences with fusion of the RID MLD and CBD to RDTND.

The *rtxA1* gene encoding the MARTX toxin is a major virulence determinant of *V. vulnificus* [37,38]. This large toxin consists of conserved N- and C-terminal regions that mediate secretion and host cell delivery, and a highly variable central region containing multiple cytopathic effector domains activated in the host cytosol (Figure 5). These domains – ACD (Actin Cross-Linking Domain), ABH (Alpha/Beta Hydrolase), RID (Rho Inactivation Domain), MCF (Makes Caterpillars Floppy-like Domain), RDTND (RID-Dependent Transforming NADase; formerly DUF1), and RRSP (RAS/RAP1-specific endopeptidase) – define the main MARTX toxin architectures previously described (types M, C, and D) [37–39]. Functionally, the toxin contributes to host colonization, tissue invasion, and, in the case of type D, triggers cytokine-storm-mediated lethality in both mouse and eel models of vibriosis [37–39].

Among the 51 L4 isolates sequenced, three *rtxA1* architectures were detected: type M (15.7%), type C (35.3%), and a previously undescribed variant (47.1%), which we designate type H (Figure 5, Supplementary Table 6). Comparative domain analysis revealed that type H carries modules characteristic of the type C and M toxins with an upstream domain showing strong similarity to the recently described RDTND of *V. vulnificus* MO6-24/O, yet with distinct sequence features suggesting excision of sequences encoding the catalytic domain of RID while retaining the RID membrane localization domain (MLD) and calmodulin binding domain (CPD) [40]. This configuration implies that, unlike reference toxins – where RDTND and RID form a single calmodulin-stabilized module – the type H toxin produces a unique RDTND with an attached MLD, although the RID MLD has been shown to have only weak plasma membrane targeting activity [41]. Based on sequence conservation and structural predictions, we propose that this domain functions as a calmodulin-dependent NADase (CdN).

**Figure 6.**
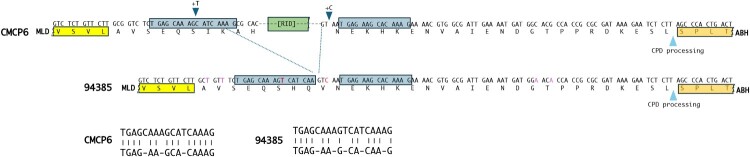
Proposed model for the evolutionary emergence of the new MARTX toxin and effector domain. The new MARTX toxin variant in strain 94385 arose from a gene nearly identical to the CMCP6 *rtxA1* gene with minor changes in codon wobble positions (marked in red). The deleted sequence in between the upstream MLD (yellow) and the downstream ABH (orange) correspond to the coding sequence for the RID catalytic domain. The region is flanked by an imperfect repeat with 14/17 bp matching (blue boxes and aligned sequences below). The domain organization variation in the new variant likely arose by an illegitimate recombination event that retained 15 bp of the upstream repeat with an inserted T nucleotide (+T) and all 14 bp of the 3’ repeat along with 4 upstream nucleotides and inserted C (+C) that likely arose from imprecise DNA repair of the DNA break. It is also possible the additional residues were acquired not in the strand break repair but rather are naturally occurring variations in the RID sequence of the parental strain.

DNA sequence alignment further revealed the presence of an imperfect 14/17 bp repeat flanking the missing *RID* coding region, indicating that the type H organization likely originated via illegitimate recombination within a *C-type rtxA1* gene. This event retained 15 base pairs (bp) of the upstream repeat plus a single-base insertion and preserved 14 bp of the downstream repeat with an additional 4 bp insertion, consistent with imprecise DNA break repair (Figure 5 and Figure 6). This mechanism explains the generation of a stable mosaic *MARTX* architecture in which C- domains coexist with a novel *CdN* effector, highlighting the dynamic nature of *rtxA1* evolution in L4.

The T6SS, a contact-dependent apparatus typical of Gram-negative bacteria, contributes to environmental fitness by mediating resistance to amoebal predation and promoting interbacterial competition [42]. Nearly all L4 isolates (98%) encoded at least one T6SS cluster, and four (7.8%) carried two distinct systems (Supplementary Table 6). The second T6SS type had been observed – but not described – in a minority of *V. vulnificus* genomes in the study by López-Pérez et al. [7]. In this work, we identified it within L4 and characterized it in detail (loci annotation with Bakta in Supplementary Table 7). The occurrence of multiple T6SS types in *Vibrio* spp. has also been reported in *Vibrio coralliilyticus*, where they are associated with distinct ecological functions – namely resistance to amoebal predation (potentially paralleling resistance to phagocytosis) and interbacterial competition [42,43].

Interestingly, both *rtxA1* and *T6SS* clusters were absent in the environmental strain 19-VB00313, which may indicate reduced competitiveness or virulence potential, but this remains a genomic hypothesis that requires experimental confirmation.

The capsule is a major determinant of environmental persistence and host infection in *V. vulnificus*. Encapsulated variants survive significantly longer than non-encapsulated forms in seawater microcosms [44] and exhibit increased resistance to serum bactericidal activity and phagocytosis [34,45].

To assess capsular diversity in L4, we analyzed the genomes containing a complete *CPS* locus (42 genomes) and compared them with reference strains for *CPS-1* (CMCP6), *CPS-2* (YJ016), and *CPS-4* (CECT 4999) [33,34,46]. None of the L4 strains corresponded to *CPS-1* or *CPS-4*; three environmental isolates matched *CPS-2*, while the remaining strains harbored previously undescribed capsule loci, defining 12 novel *CPS* genotypes. Genotype 5 was the most prevalent (17.6%, including one clinical isolate), and genotypes 10 and 11 shared transport genes with *CPS-2*. Several loci contained adjacent transposase genes, suggesting possible involvement of transposon activity in capsule diversification (Supplementary Figure 2).

Together, the diversity of *MARTX, T6SS*, and *CPS* genotypes demonstrates that L4 exhibits exceptional virulence-associated variability, consistent with frequent recombination and genomic plasticity that may facilitate adaptation to diverse hosts and environments**.**

### Mobile genetic elements and multi-host potential

Prophage analysis revealed that 21 of the 51 L4 genomes (41.2%) contained at least one prophage region, most commonly belonging to *Caudoviricetes, Corticoviridae, or Inoviridae* families. Prophage content ranged from 5.4 to 68.2 kb per genome, with one to three regions per strain (Supplementary Table 6). These regions encoded typical structural and integration genes but no known virulence determinants. The heterogeneous distribution of prophages among isolates suggests ongoing exchange of mobile elements that contribute to L4 genomic plasticity.

Putative plasmid elements were detected in two isolates (18-VB00627 and 20-VB00250), although plasmids could not be fully assembled due to genome fragmentation. Conjugative plasmid genes were found in both, but only strain 20-VB00250 harbored the fish-associated virulence genes *ftbp* and *fpcrp*. These encode an outer-membrane protein and an outer-membrane lipoprotein that confer host-specific resistance to fish innate immunity [28, 34]. Although absent from other L4 genomes, their loss during sequencing cannot be excluded, as *pVir* plasmids occur at very low copy number [10].

The detection of *ftbp* and *fpcrp* in L4 provides the first evidence that this lineage can acquire fish-associated virulence determinants via horizontal gene transfer.

### Functional assays: resistance to human serum and antimicrobial susceptibility

To evaluate the clinical relevance of the genomic findings, selected L4 strains were tested for survival in human serum supplemented or not with iron to simulate elevated iron levels, a major risk factor for sepsis in *V. vulnificus* infections [8,9]. Antibiotic susceptibility was also assessed.

Most of the selected strains (80%) survived in human serum (percent survival > 60%), and all serum-resistant strains were able to multiply when iron was added, increasing their population size more than 500-fold in just 4 h (Table 1). Iron-overloaded human serum mimics the condition that predisposes individuals to *V. vulnificus*-induced sepsis.

Capsular genotype comparison revealed that serum resistance was not restricted to CPS-1, CPS-2*,* or CPS-4 serotypes [33,34] but also occurred in strains with novel *CPS* loci, and therefore, novel CPS serotypes (Table 1).

The *ftbp/fpcrp*-positive strain was tested in eel serum and found to be sensitive (< 60% survival) [11]. This negative result is compatible with the hypothesis that these genes may confer virulence in other fish species, which warrants further testing.

Antimicrobial susceptibility testing showed that most L4 isolates (all of them pre-2018) remain susceptible to frontline therapeutics such as fluoroquinolones, carbapenems, and third-generation cephalosporins. Ampicillin resistance was common, while tetracycline, chloramphenicol, and aminoglycoside resistance were rare (Supplementary Table 4). Genomic screening with ABRicate identified only three antibiotic resistance genes (*blaCARB-17*, *tet*[*35*], *catB9*), all of which were present in all genomes. One of them *blaCARB-17* was clearly associated with ampicillin resistance; however, in the other cases, we found no correlation between genetic content and resistance phenotype.

Collectively, these results show that a significant proportion of L4 isolates can withstand and even proliferate in iron-overloaded serum, a trait associated with the risk of sepsis in susceptible individuals, while remaining sensitive to key antimicrobials. These findings underscore the clinical relevance of L4 as a potential opportunistic pathogen in warming European waters and highlight its multi-host, climate-responsive adaptive profile**.**

## Discussion

*V. vulnificus* is a severe and emerging public health threat in temperate regions, particularly affecting vulnerable populations during warm months [1,14]. Although human infections have traditionally been associated with tropical and subtropical areas, recent increases in SST have facilitated the expansion of pathogenic *Vibrio* species into higher latitudes [14,16,47]. All isolates analyzed in this study were collected before 2018, providing a historical snapshot of the lineage composition and geographical distribution prior to the recent acceleration of SST rise and *Vibrio* incidence across Europe. Therefore, this study offers a retrospective, genomics-based view of *V. vulnificus* L4 and expands our understanding of how environmental change and horizontal gene flow shape the evolution of this zoonotic marine pathogen.

The detection of L4 in German coastal waters – previously known only from the western Mediterranean – reveals a clear historical signal of climate-linked northward spread. These findings strengthen the evidence that warming seas are driving the poleward expansion of *Vibrio* populations in European waters. Within this context, the absence of L3 among Baltic isolates is particularly noteworthy. Both L3 and L4 originated in the Mediterranean basin [5,6,48], yet only L4 was detected in northern samples. This suggests that ecological or genetic traits specific to L4 – such as enhanced tolerance to low salinity or greater environmental persistence – may have favoured its establishment under temperate conditions. Continued monitoring will be essential to determine whether this distribution pattern reflects selective adaptation or sampling bias.

The detection of the newly identified L4 isolates was enabled by a multiplex PCR assay specifically designed to detect Mediterranean-associated lineages. Combining previously validated markers for species- and L3-level detection [28] with a novel L4-specific marker identified through comparative pangenome analysis, this assay proved fully concordant with phylogenomic assignment and offers a robust tool for future surveillance and ecological monitoring of this European lineage.

Beyond detection, comparative genomics revealed remarkable variability in key virulence and survival determinants, reflecting the high genomic plasticity of L4. This variability encompasses multiple structural variants of the multifunctional *rtxA1* toxin gene, fourteen distinct capsular genotypes, and two T6SS loci. Together with the heterogeneous distribution of prophages, these features illustrate how structural and mobile elements jointly sustain the adaptive flexibility of L4.

The structural diversity of MARTX toxins in *V. vulnificus* arises from frequent recombination between homologous or short repeated sequences flanking effector-domain regions – a process that generates chimeric toxins with new functional configurations [11,37,39]. In this study, we identified a novel MARTX variant – designated type H – in nearly half of all L4 isolates. Comparative domain mapping and sequence boundary analysis suggest that type H emerged through illegitimate recombination between short repeats within a *type C rtxA1* gene, resulting in both a new modular arrangement and a unique upstream effector domain. This domain has the RDTND catalytic domain fused with the downstream CBD and MLD from RID. This new effector would create a calmodulin-dependent NADase (CdN) that depletes host cytosolic NAD^+^, but in the absence of RID activity that can inactive Rho GTPases at the plasma membrane to suppress inflammatory gene expression [49]. This newly organized effector CdN may not localize to the membrane as the RID MLD is known to have only weak plasma membrante targeting actvity [41].

Such uncoupling between CdN and RID activities could lead to deregulated cytopathic outcomes, potentially enhancing the overall virulence or adaptability of the bacterium. Functionally, this configuration broadens the MARTX toxic potential beyond the classical C and M types – largely associated with colonization and invasion – and approaches the heightened cytotoxic profile described for type D, which can trigger cytokine storms in vivo [36]. The emergence of this new variant underscores the evolutionary plasticity of *V. vulnificus*, illustrating how micro-recombination and imprecise DNA repair events in aquatic environments can give rise to novel effector architectures with expanded or altered functions.

Capsular diversity also appears central to L4 adaptation. Variation in surface polysaccharides is a classical bacterial strategy to evade bacteriophage predation – one of the dominant selective pressures in aquatic ecosystems [45] – and encapsulated variants survive significantly longer than non-encapsulated forms in seawater microcosms [44]. Encapsulation additionally enhances resistance to serum bactericidal activity and phagocytosis [34,45]. Our data extend the repertoire of serum-resistant CPS beyond canonical types (CPS-1, CPS-2, CPS-4): several novel L4 genotypes also conferred resistance to human serum, indicating that multiple, previously unrecognized CPS configurations can mediate serum survival. We hypothesize that the frequent presence of transposase genes adjacent to CPS loci supports the view that transposon activity contributes to capsule diversification, reinforcing the capsule as both an environmental persistence factor and a virulence determinant.

Additional evidence of diversification comes from the detection of plasmid-borne conjugative and virulence genes *(ftbp and fpcrp)* in two L4 isolates. These virulence genes, transferable by conjugation, confer resistance to innate immunity in fish blood and are under positive selection [11]. The L4 strain carrying both genes was serum-sensitive in eel assays, suggesting a different natural host, but their presence confirms that L4 can integrate new virulence factors via HGT, potentially expanding its host range and reinforcing its zoonotic character. Such acquisitions parallel events in *V. cholerae* and *V. harveyi,* where HGT drives the emergence of new virulence or resistance traits [50,51].

From a clinical standpoint, most L4 isolates remain susceptible to frontline antibiotics, supporting the continued effectiveness of current therapies [52]. Ampicillin resistance was common but consistent with previous reports [53]. Although L4 clinical isolates were relatively few (≈11.8%), their ability to resist and proliferate in iron-enriched human serum suggests potential to cause sepsis in susceptible individuals. Continuous genomic and clinical surveillance will be essential to monitor any increase in L4-associated infections.

From a public-health perspective, the detection of a lineage previously restricted to the Mediterranean in northern European waters has major implications. Raw or undercooked seafood consumption and wound exposure remain the principal infection routes [2,16,20]. Despite the rising number of *Vibrio* infections in temperate zones, *Vibrio* spp. remain excluded from EU food-safety microbiological criteria (Regulation (EC) No 2073/2005), and no EU-wide notification system exists for human infections. This regulatory gap contrasts with national initiatives such as in Germany, where *Vibrio* infections became notifiable in 2021 [19]. Given the high case-fatality rate among at-risk individuals – often exceeding 50% even with treatment [1] – early detection and risk mitigation are crucial. The L4-specific multiplex PCR developed here provides a lineage-specific tool for both environmental and clinical surveillance, supporting One Health monitoring of *V. vulnificus* in a warming Europe.

In conclusion, this study provides the first comprehensive description of a European lineage (L4) of *V. vulnificus* that has diversified under climate-driven northward expansion. L4 now encompasses strains with mosaic virulence architectures, novel capsule genotypes, and horizontally acquired fish-pathogenic genes, reflecting a convergence of environmental adaptation, genetic exchange, and ecological opportunity. Similar temperature-driven range shifts have been documented for *V. cholerae* and *Vibrio parahaemolyticus* [14], but unlike these globally distributed species, L4 represents a regional lineage undergoing early-stage ecological diversification within Europe. One L4 strain also harboured the plasmid-borne genes *ftbp* and *fpcrp*, which confer resistance to fish innate immunity and the ability to cause sepsis, thereby extending the distribution of the *piscis* pathovar to all five *V. vulnificus* lineages. Collectively, these findings reinforce that *V. vulnificus* is a multi-host, zoonotic, and climate-responsive pathogen, whose continued diversification exemplifies how ocean warming and horizontal gene flow jointly drive the evolution of high-risk marine bacteria within a One Health framework.

The predominance of isolates from northern European waters and their demonstrated ability to resist and proliferate in iron-enriched human serum – probably related to their capacity to cause sepsis in susceptible individuals – indicate that L4 is a newly adapted, climate-associated lineage bridging environmental, animal, and human health under a One Health framework.

## Supplementary Material

Supplementary_table_3.xlsx

Supplementary_table_6.xlsx

Supplementary_Figure 2.png

Supplementary_table_5.xlsx

Supplementary_table_7.xlsx

Supplementary_Table2.xlsx

Supplementary_table_1.xlsx

Supplementary_Figure 1.png

Supplementary_table_4.xlsx

## Data Availability

The data that support the findings of this study are openly available in GenBank under BioProject ID PRJNA1287076.
