# Supplementary figures and images for: Emergence, climate-driven expansion, and diversification of a European Vibrio vulnificus lineage (L4) with multi-host pathogenic potential
Source: Emerg Microbes Infect. 2025 Dec 9;15(1):2601370. doi: 10.1080/22221751.2025.2601370 (PMC12777771; doi:10.1080/22221751.2025.2601370)

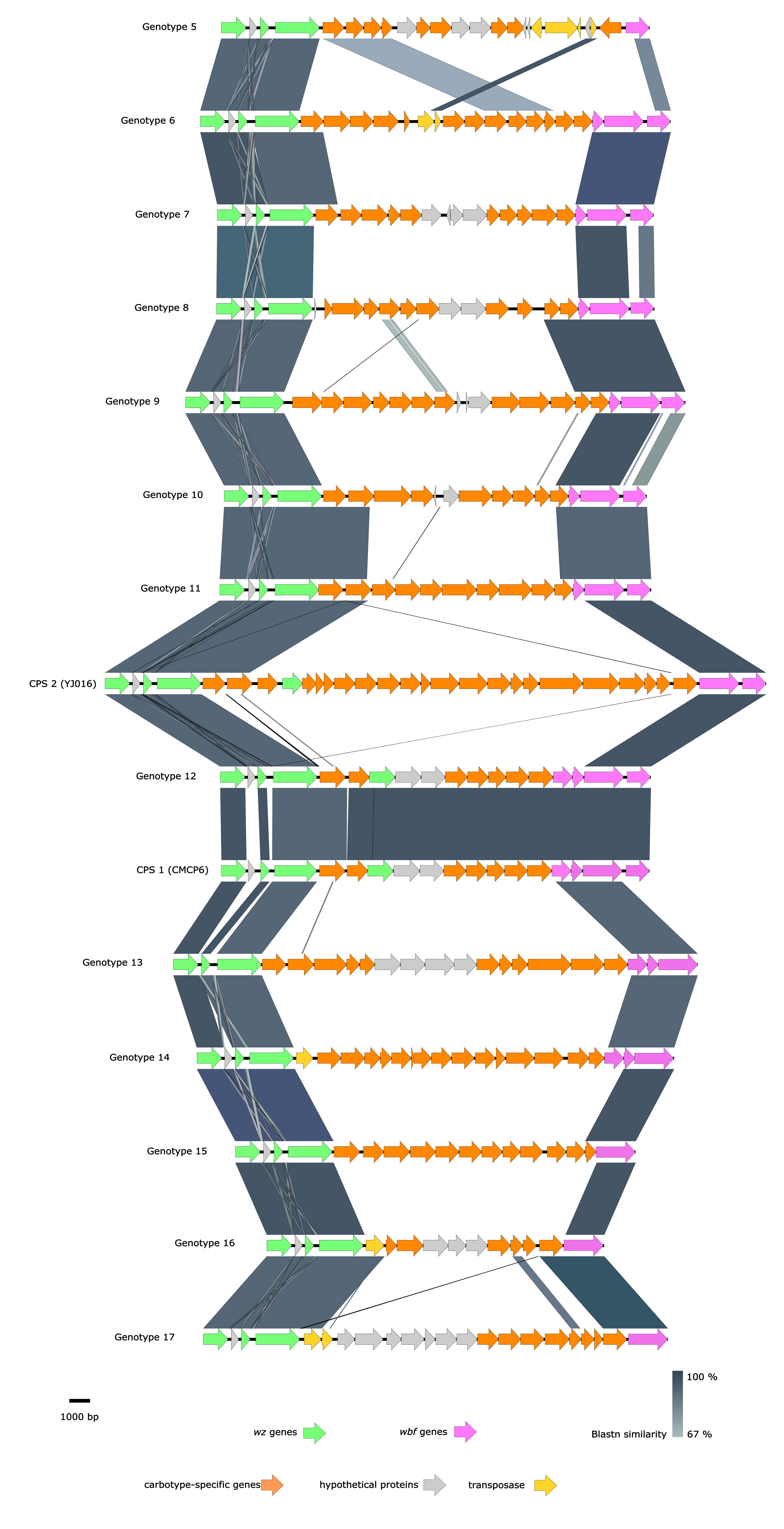

Supplement: Supplementary_Figure 2.png [file TEMI_A_2601370_SM8504.png]

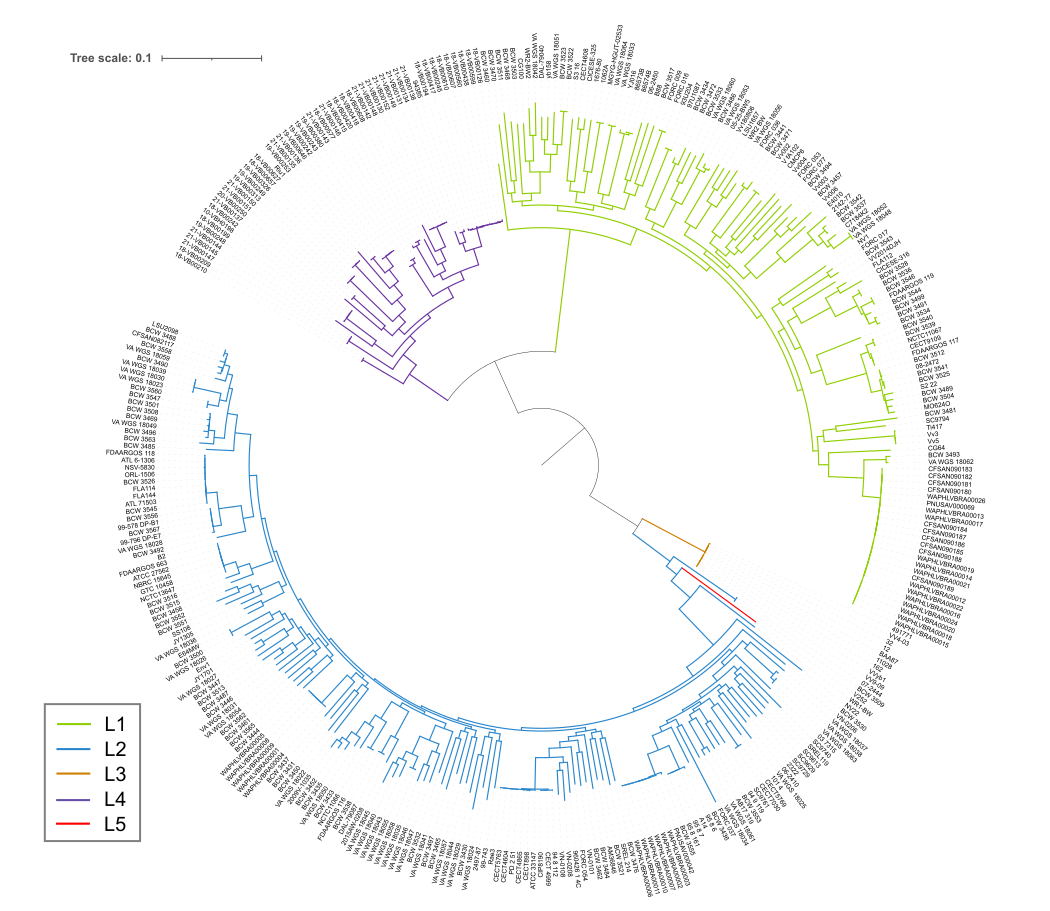

Supplement: Supplementary_Figure 1.png [file TEMI_A_2601370_SM8499.png]
